# Real-world validation of a length prediction formula for bedside PICC placement: A single-center retrospective observational study

**DOI:** 10.1371/journal.pone.0349256

**Published:** 2026-05-14

**Authors:** Jung Won Kwak, Sung-Joon Park, Hwan Hoon Chung

**Affiliations:** 1 Department of Radiology, Korea University College of Medicine, Korea University Anam‌‌ Hospital, Seoul, South Korea; 2 Department of Radiology, Korea University College of Medicine,‌‌ Korea University Ansan Hospital, Ansan-si, Gyeonggi-do, South Korea; Baylor College of Medicine, UNITED STATES OF AMERICA

## Abstract

This study aimed to validate a landmark-based catheter length prediction formula for bedside peripherally inserted central catheter (PICC) placement in a real-world clinical setting, especially in complex environments where advanced navigation technologies may be limited or unsuitable. We retrospectively analyzed 449 consecutive bedside PICC placements performed between March 2022 and September 2025. Catheter insertion length was estimated beforehand using a formula based on anteroposterior chest radiograph (CXR) measurements, including clavicle length and vertebral body units. Final tip positions were assessed by two independent radiologists using post-procedural CXR in relation to the cavoatrial junction (CAJ), with optimal positioning defined as the tip being within ±2.8 cm of the CAJ. Of the 449 cases, technical success was achieved in 436 (97.1%), and optimal tip positioning was seen in 74.3% of successful cases. The interobserver agreement for tip assessment was 98.45% (95% confidence interval: 96.84–99.25%). The average distance from the catheter tip to the CAJ was −0.10 ± 2.59 cm, with a median of 0.0 cm. Rates of optimal positioning did not significantly differ based on operator experience (p = 0.496) or approach side (p = 0.590). Importantly, the technical success rate remained high (97.5%) even in patients with other intravascular devices, such as extracorporeal membrane oxygenation (ECMO) cannulas or hemodialysis catheters. In conclusion, the landmark-based formula offers high predictive accuracy and excellent reproducibility for bedside PICC placement. It serves as a reliable initial safety measure and a practical alternative in complex intensive care settings where real-time navigation is limited or systemically constrained.

## Introduction

Peripherally inserted central catheters (PICCs) are increasingly used for durable venous access in patients receiving chemotherapy, prolonged antimicrobial therapy, or parenteral nutrition. Compared with conventionally placed central venous catheters (CVCs), PICCs can be used for longer-term placement with a lower risk of immediate mechanical complications, and are suitable for ambulatory care [1,2]. The safety and effectiveness of PICCs are correlated with accurate tip placement; malposition is associated with arrhythmias, venous thrombosis, and suboptimal drug delivery [3,4].

The cavoatrial junction (CAJ) is widely accepted as the preferred target for PICC tip placement because it balances function with reduced vascular injury [3]. In angiography suites, fluoroscopic guidance generally eliminates uncertainty about catheter length. By contrast, when patients cannot be transferred—because of hemodynamic instability, extracorporeal membrane oxygenation (ECMO) cannulation, or continuous renal replacement therapy—PICC insertion is performed at the bedside. While current international guidelines strongly recommend intra-procedural tip location using intracavitary electrocardiography (IC-ECG) or ultrasound to ensure accuracy, these technologies face practical hurdles in diverse clinical settings. IC-ECG, for instance, requires specialized equipment and a stable cardiac rhythm to identify P-wave changes; however, many intensive care unit (ICU) patients may have pre-existing arrhythmias, pacemaker leads, or interference from extracorporeal membrane oxygenation (ECMO) circuits that can render IC-ECG signals unreliable or impossible to interpret. In cases where surgical procedures such as sternotomy have been performed or complex life support devices have been installed, the use of echocardiography is also limited because it hinders direct skin contact, thereby obscuring the acoustic window required for imaging.

Furthermore, the availability of advanced navigation systems, such as IC-ECG or real-time fluoroscopy, is often limited by economic and infrastructural factors, as well as regulatory hurdles and supply chain logistics in many regions. In several countries, these specialized devices are not yet widely distributed or remain unavailable in standard wards and emergency settings due to prolonged governmental licensing processes or insufficient distributional networks. In these situations, anthropometric or landmark-based length prediction methods are required. In such environments, a robust pre-procedural formula serves as a critical initial safety measure to reduce the risk of malposition when advanced real-time guidance is inaccessible. Operators typically estimate catheter length (ECL) from bony and soft-tissue landmarks and the patient’s height before placement, then confirm tip position using postprocedural chest radiography (CXR) [5–9].

Against this backdrop, Lee et al. introduced a landmark-based formula to predict insertion length using anteroposterior (AP) chest radiograph (CXR) metrics [10]. Although the method showed encouraging accuracy in a controlled cohort, external validation in complex, multi-device clinical environments—where tip malposition risk is highest—is needed [11,12]. This study aimed to validate the formula proposed by Lee et al. under real-world clinical conditions, specifically assessing its accuracy and reproducibility across operators in a high-complexity clinical population.

## Materials and methods

### Ethics statement

The study was approved by the Institutional Review Board of Korea University Ansan Hospital (IRB No. 2025AS0263). The requirement for informed consent was waived by the board due to the retrospective nature of the study, and all procedures were performed as part of routine clinical care. Data were accessed for research purposes on October 1, 2025. During and after data collection, the authors had no access to information that could identify individual participants, as all data were fully anonymized before analysis.

### Study design and setting

This study was a single-center, retrospective observational study of consecutive adults undergoing bedside PICC placement in routine clinical practice. Bedside procedures were prioritized for patients unable to be transferred to the angiography suite because of hemodynamic instability or competing devices (e.g., ECMO cannulas, continuous renal replacement therapy circuits).

### Participants

Eligible patients were ≥18 years and required bedside PICC placement for chemotherapy, prolonged antimicrobial therapy, or parenteral nutrition. Exclusion criteria were incomplete imaging confirmation, known central venous anomalies precluding standard tip assessment (including persistent left superior vena cava, situs inversus, and other congenital variants affecting central venous anatomy, such as those found in adults with congenital heart disease), or immediate procedural failure (wire/catheter nonadvancement or abandonment).

### Length estimation (landmark-based formula)

Catheter insertion length was determined a priori using the landmark-based formula reported by Lee et al., without modification [10]. The method calculates a predicted length from anteroposterior (AP) CXR measurements together with the patient’s sex and approach side.

**Fig 1:**
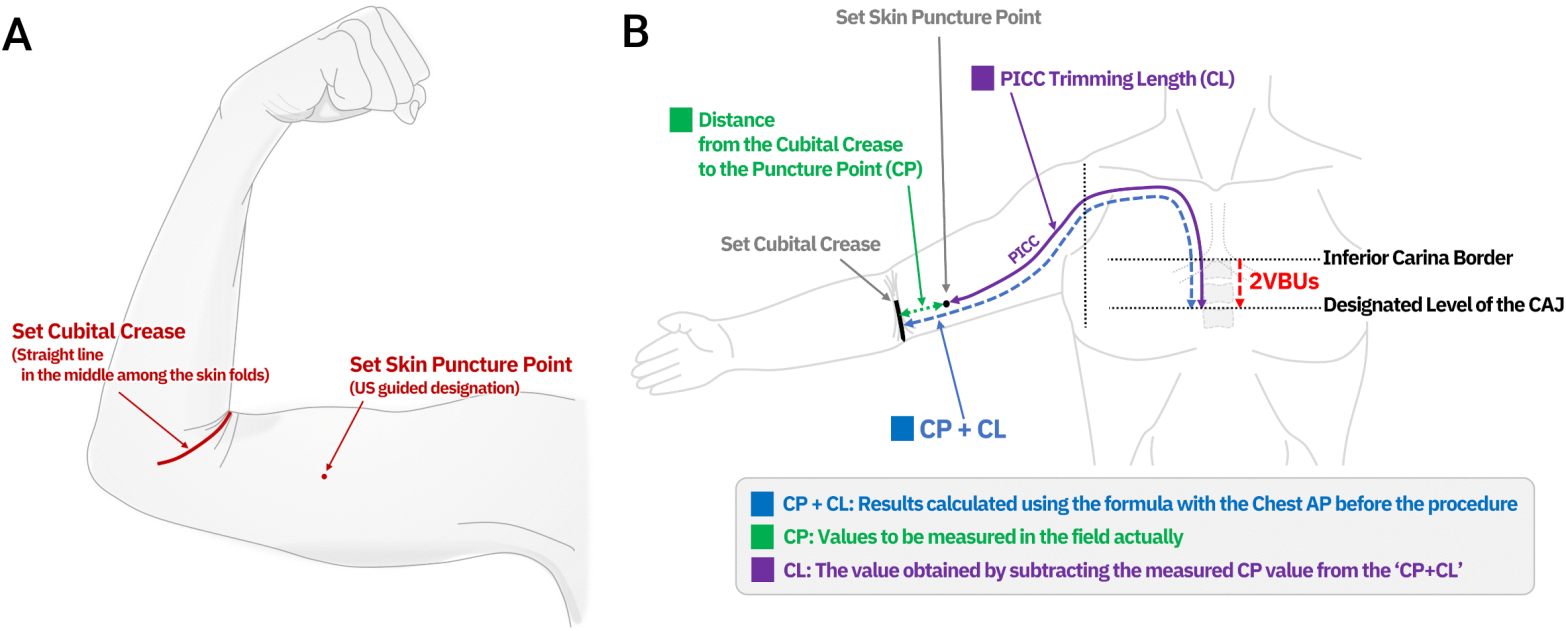
Schematic illustration of the landmark-based PICC length calculation method. **(A)** Anatomical landmarks identified at the bedside: the cubital crease (established by flexing the elbow to 90°) and the skin puncture point (determined by ultrasound guidance). **(B)** The formula calculates the total length (CP + CL), which represents the distance from the cubital crease to the CAJ. At the procedure site, the operator measures the actual distance from the cubital crease to the puncture point (CP, green line). The catheter trimming length (CL, purple line) is then determined by subtracting CP from the precalculated total length (CP + CL). The catheter is trimmed to CL before insertion, ensuring the tip reaches the designated CAJ level (defined as 2 VBU below the inferior carina border). PICC: peripherally inserted central catheter; CP: distance from cubital crease to puncture point; CL: catheter length; CAJ: cavoatrial junction; VBU: vertebral body unit.


The final ECL was established by measuring the distance from the cubital crease to the skin puncture site (CP). This study adhered to the published measurement sequence and calculation rules. CP determination is fundamental for accurate bedside PICC placement. The cubital crease was identified by flexing the elbow to 90° and marking the midline skin fold. After ultrasound designation of the puncture site, CP was measured as the shortest distance between the two marks. ECL was calculated by subtracting CP from the total predicted length by the formula; the catheter was trimmed to this length before insertion (Fig 1). The applied formula was:

$$\begin{array}{c} CP+ECL=19.831-0.062\times cCL+0.255\times 2RHD+0.720\times HVD+0.761\times TCD+1.024\times 2VBU. \end{array}$$

* cCL: contralateral clavicle length (cm), *2RHD: 2nd ribs horizontal distance (cm), *HVD: ipsilateral humero-vertebral distance (cm), *TCD: ipsilateral thoraco-carinal distance (cm), *2VBU: vertical distance of 2 vertebral body units (cm), *Note: For left-sided approach, add 2.843 cm; for female patients, subtract 0.821 cm.

### Catheter insertion

All procedures were performed by four interventional radiologists with 1, 3, 9, and ≥20 years of experience. Procedures used maximal sterile precautions and ultrasound-guided venous access (microintroducer technique). With the ultrasound probe positioned over the ipsilateral internal jugular vein (IJV), operators verified that the guidewire did not enter the IJV; if the tip was visualized in the IJV, the guidewire was withdrawn and redirected. Notably, ultrasound was used exclusively for venous access guidance and IJV monitoring—not for real-time catheter tip navigation or tip position confirmation. The pretrimmed PICC catheter was inserted using an over-the-wire technique. All catheters were 5-Fr, dual-lumen PICC sets (Power PICC; BD Biosciences, Salt Lake City, UT, USA). The device was advanced to the pretrimmed length; no fluoroscopic guidance was used for bedside cases.

### Tip confirmation and definitions

**Fig 2:**
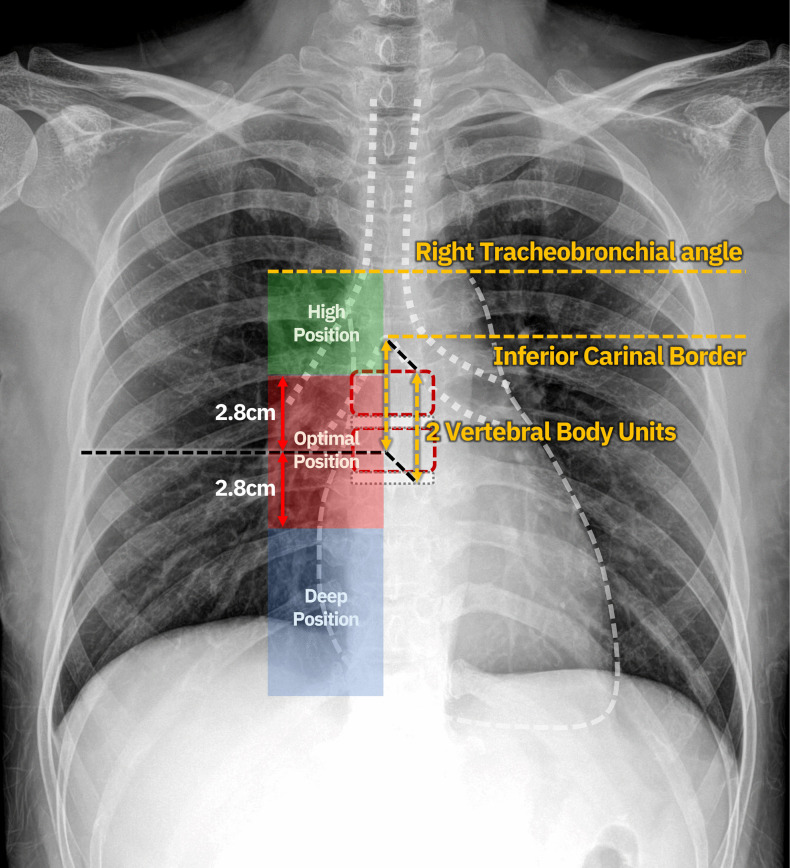
Definition of optimal, high, and deep PICC tip positions on anteroposterior chest radiography. The cavoatrial junction (CAJ) is defined as 2 VBU below the inferior carina border. Optimal position (red zone) is the catheter tip located within ±2.8 cm of the designated CAJ. High position (green zone) indicates the tip located in the superior vena cava between the optimal zone and the right tracheobronchial angle. Deep position (blue zone) indicates the tip located in the right atrium below the optimal zone. CAJ: cavoatrial junction; VBU: vertebral body unit.

Final tip position was verified after placement using AP-CXR. Radiographic assessment used carinal or equivalent thoracic landmarks as surrogates for the CAJ (2VBU below the inferior carina border), following established methods [10]. Two interventional radiologists (9 and 7 years of post-fellowship experience, respectively) independently reviewed each AP-CXR; discrepancies were resolved by consensus. The primary endpoint was absolute distance (mm) from the catheter tip to the CAJ reference. Tip location was categorized as optimal, high, or deep, according to prior literature and institutional policy (Fig 2) [10].

(1) Optimal: tip within ±2.8 cm of the designated CAJ on AP-CXR.
(2) High (proximal to target): tip within the superior vena cava (SVC) zone, specifically between the superior border of the optimal zone and the upper margin of the SVC at the right tracheobronchial angle.
(3) Deep (distal to target): tip below the inferior border of the optimal zone, within the right atrium (RA).
(4) Technical failure: abnormal insertion (e.g., into the IJV, azygos vein, or contralateral brachiocephalic vein) or intravascular rolling. Intravascular rolling was defined as the catheter or guidewire curling back upon itself (forming a U-shaped loop) within the peripheral or proximal venous lumen, failing to advance into the central venous system. This phenomenon typically occurs at or near the axillary or subclavian vein and was identified on post-procedural chest radiography by a redundant, looped catheter course.
(5) Anatomic failure: inability to complete PICC insertion because of vascular anatomy (occlusion, severe stenosis, hostile angulation), resulting in termination with a midline catheter.

### Reproducibility across operators

Operator identity and experience were recorded. Reproducibility was assessed by comparing absolute CAJ distance across operators and quantifying interoperator variability.

### Statistical analysis

Statistical analyses were performed using SPSS (version 25.0; IBM Corporation, Armonk, NY, USA) [13]. Continuous variables were analyzed as mean ± standard deviation (SD) or median (interquartile range), as appropriate, and compared using *t*-tests, ANOVA (analysis of variance), or nonparametric equivalents. Categorical variables were compared with χ^2^ or Fisher’s exact tests. Interoperator reproducibility was evaluated using one-way ANOVA on absolute CAJ distance and an intraclass correlation coefficient (two-way random effects, absolute agreement). Two-sided *p* < 0.05 was considered statistically significant.

## Results

### Study flow

From March 2022 to September 2025, 459 bedside PICC candidates were identified. Ten patients were excluded because of anatomic issues requiring conversion to a midline catheter. The remaining 449 attempts comprised the analytic cohort and were categorized as optimal (*n* = 324, 72.2%), suboptimal (*n* = 112, 24.9%; SVC *n* = 59, RA *n* = 53), or technical failure (*n* = 13, 2.9%). The technical success rate (excluding technical or anatomic failures) was 436/449 (97.1%). No patients with known congenital heart disease affecting central venous anatomy were identified in the study cohort.

### Interobserver agreement

There were 7/452 (1.55%) discordant readings; crude agreement was 98.45% (95% confidence interval [CI]: 96.84–99.25%; Wilson). Relative to high-agreement thresholds, observed agreement exceeded 0.90 (one-sided exact binomial *p* = 3.68 × 10^−13^) and 0.80 (*p* = 7.47 × 10^−34^).

### Optimal insertion by the operator and the access side

Operator caseloads and optimal rates were: A (*n* = 232; 71.6% optimal), B (*n* = 21; 61.9%), C (*n* = 72; 77.8%), and D (*n* = 124; 71.8%). A 2 × 4 χ^2^ test found no significant difference in optimal rates among operators (*p* = 0.496).

Right-sided approaches were performed in 408 cases (90.9%), with optimal position in 296 (72.5%). Left-sided approaches were performed in 41 (9.1%), with optimal position in 28 (69.3%). Fisher’s exact test showed no significant difference (*p* = 0.590).

### Tip location relative to the CAJ

**Fig 3:**
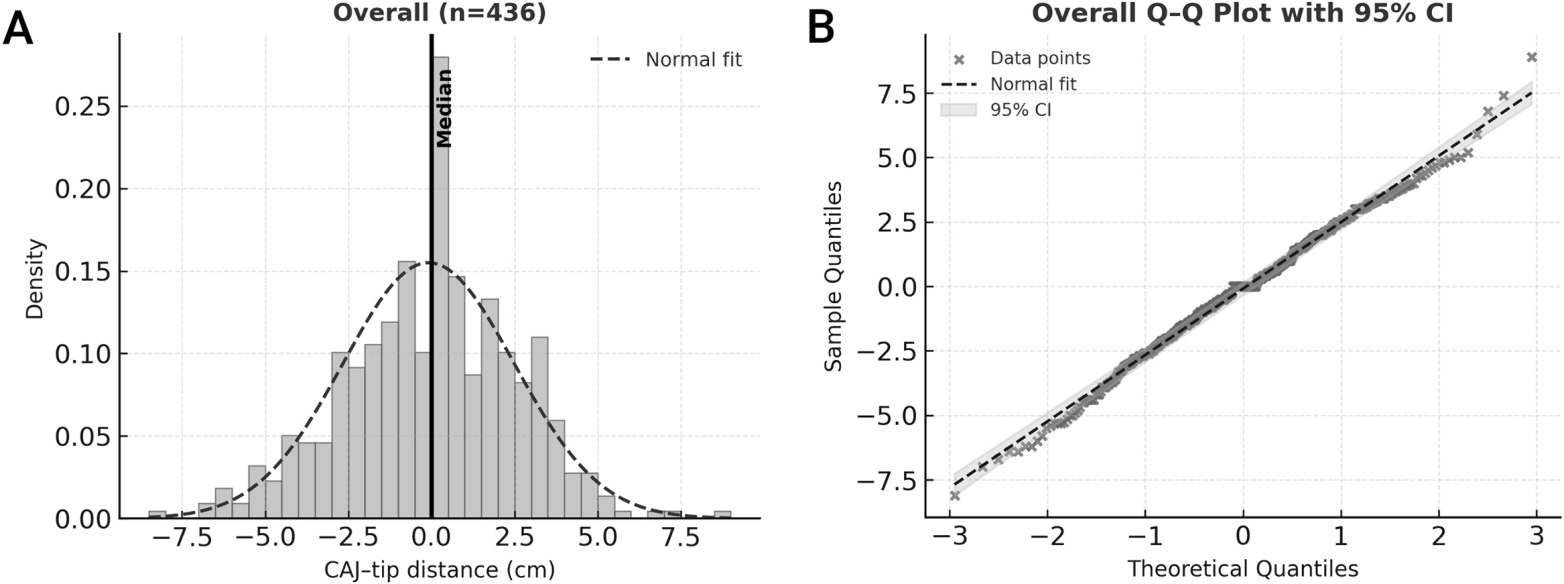
Distribution of catheter tip-to-cavoatrial junction (CAJ) distances. **(A)** Histograms of catheter-tip distributions for all bedside PICC procedures. The distribution is approximately symmetric around a median of 0 cm from the designated CAJ. **(B)** Q–Q plot of catheter tip-to-designated CAJ distance for all bedside PICC procedures, demonstrating an approximately normal distribution.

Across all evaluable insertions, the CAJ–tip vertical distance was centered near zero (median, 0.0 cm; mean, −0.10 ± 2.59 cm; minimum, −8.1 cm; maximum, + 8.9 cm) (Fig 3A).

**Fig 4:**
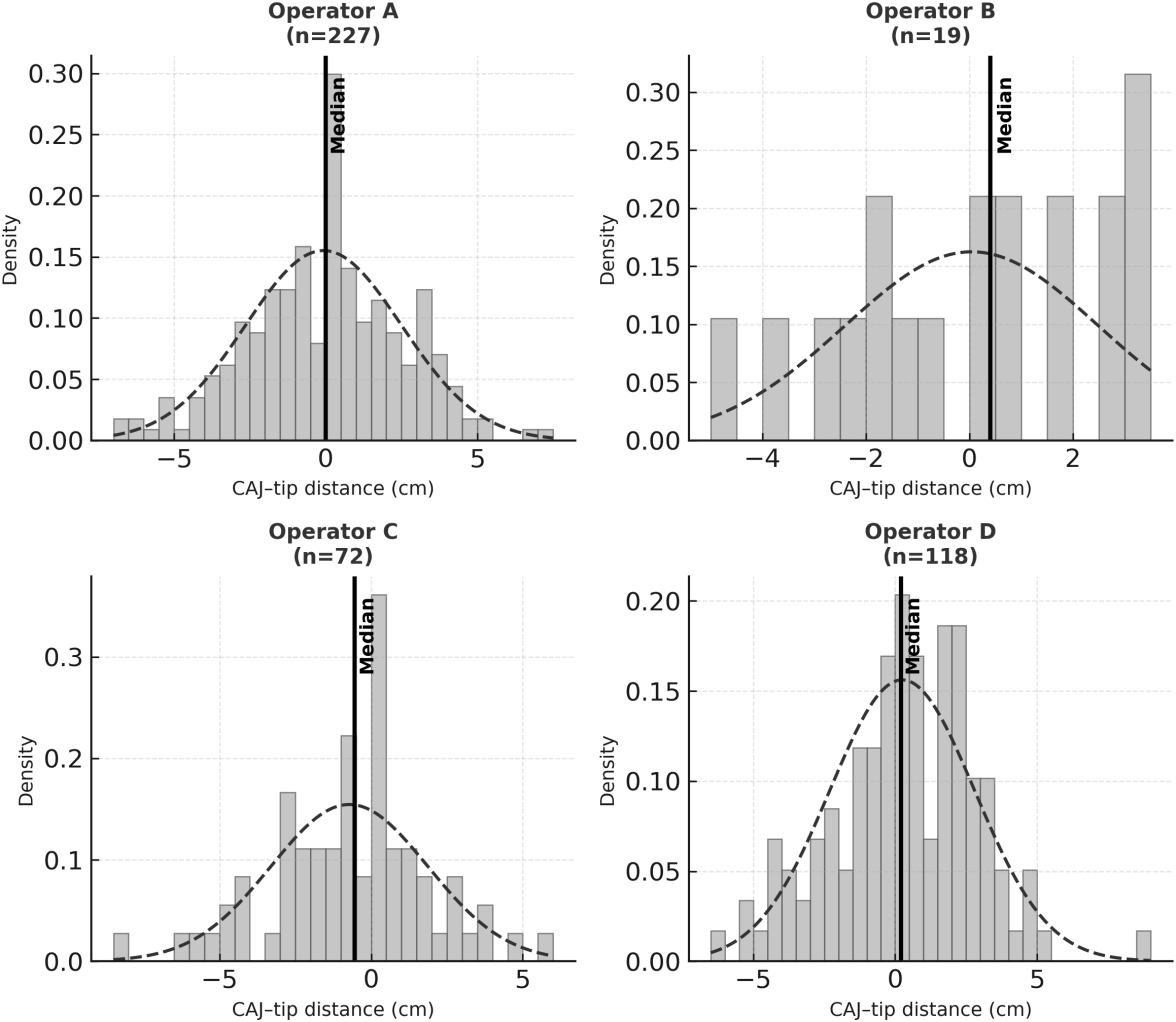
Operator-specific histograms of catheter-tip distributions. With the exception of operator B (*n* = 19), whose sample size is very small, tips are positioned approximately symmetrically around a median of 0 cm from the designated CAJ.

Distributional tests supported approximate normality (Shapiro–Wilk *p* = 0.248; D’Agostino–Pearson *p* = 0.391; Anderson–Darling: normal). In the Q–Q plot, points lay close to the diagonal, consistent with normality (Fig 3B). By operator, median (interquartile range) absolute CAJ distance was: A, 1.78 cm (1.01–3.02); B, 1.97 cm (1.14–2.97); C, 1.50 cm (0.80–2.80); D, 1.70 cm (0.95–2.80). Operator-stratified tests were nonsignificant in each stratum; for the smallest stratum (*n* = 19), inference should be interpreted cautiously given limited power (Fig 4). A Kruskal–Wallis test showed no significant difference in distance distributions across operators (*p* = 0.089).

To evaluate the clinical utility of the PICC CL–prediction formula applied in this study, we analyzed 436 bedside placements (optimal, 324; suboptimal, 112) performed by four independent operators. Operator-specific SDs of the absolute CAJ–tip distance clustered tightly between 2.455 and 2.583 cm (coefficient of variation, 1.9%), indicating very low variability. Operator-specific means ranged from −0.719 to 0.247 cm, yielding a maximum interoperator difference of <1 cm. For the entire cohort, the 95% CI of the mean CAJ–tip distance was [−0.328, 0.155] cm, indicating placements were very close to the CAJ target. The overall 2-SD range ([−5.235, 5.062] cm) was similar to operator-specific ranges, demonstrating excellent reproducibility and consistent predictive accuracy irrespective of operator experience, technical nuances, or anatomic variability.

### Concomitant intravascular devices on CXR

Additional vascular devices were present in 202/449 cases (45.0%). Across those patients (multilabel counting), device mentions were: CVC, 47.9%; hemodialysis catheters, 29.8%; ECMO, 14.3%; chemoport, 5.0%; pacemaker leads, 2.9%.

**Fig 5:**
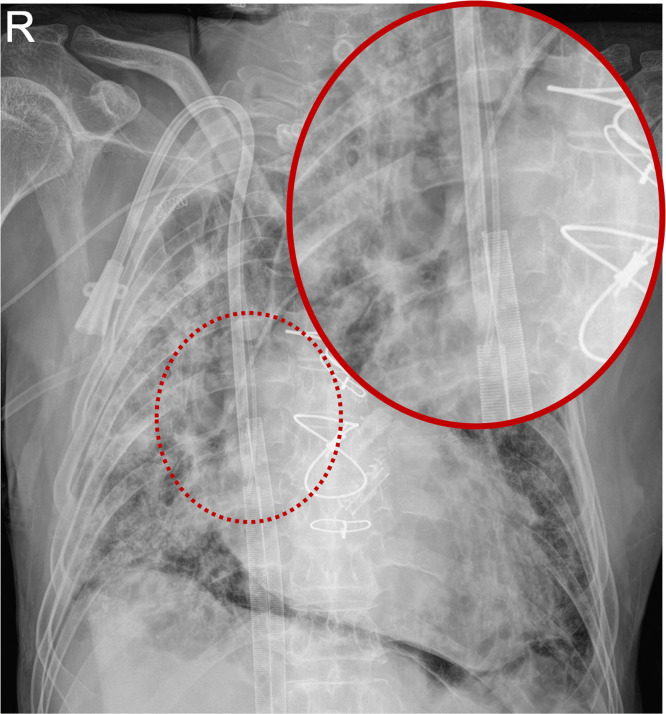
Bedside PICC placement in a complex clinical environment with multiple concomitant intravascular devices. Representative chest radiograph showing the coexistence of a same-side Permcath, a contralateral PICC, and two femoral extracorporeal membrane oxygenation (ECMO) sheaths. Despite the high clinical complexity and potential for device interference, the catheter tip is accurately localized at the target zone using the pre-procedural length prediction formula.

In the patient with the greatest number of catheters, a same-side Permcath, a contralateral PICC, and two femoral ECMO sheaths were present; the contralateral PICC tip lay within the ECMO sheath, and the Permcath tip was within 1 cm of the sheath (Fig 5).

Technical success did not differ by the presence of a concomitant intravascular device: among 201 bedside cases with another device, technical success was achieved in 197 (97.5%); among 248 without other devices, success was achieved in 237 (95.6%). Although numerically higher in the device group, the difference was not statistically significant (Fisher’s exact test: odds ratio, 1.83; 95% CI: 0.62–5.35; *p* = 0.314).

## Discussion

### Principal findings

In this single-center, real-world validation of the AP-CXR–based planning formula reported by Lee et al., bedside PICC placement achieved a technical success rate of 97.1%, with 74.3% optimal placements among technically successful cases, excellent interobserver agreement (98.45%), and no significant differences by operator or laterality. The population distribution of CAJ–tip distance was centered at 0.0 cm, and absolute distances were comparable across operators. These results demonstrate that the formula provides high precision and reproducibility, even in a diverse clinical cohort where advanced real-time navigation might be limited.

### Addressing the clinical necessity: Beyond the “Gold Standard”

Current international guidelines strongly advocate for intra-procedural tip location using intracavitary electrocardiography (IC-ECG) or ultrasound-based methods. While these technologies are considered the gold standard, our findings suggest that a robust length-prediction formula remains an essential “safety buffer” rather than an obsolete practice. In many intensive care units (ICUs), patients are frequently in states—such as severe arrhythmia, presence of pacemaker leads, or electrical interference from extracorporeal membrane oxygenation (ECMO) circuits—that render IC-ECG signals unreliable or impossible to interpret. In such high-acuity “real-world” scenarios, relying solely on real-time navigation without a pre-procedural plan can increase the risk of initial malposition. Our approach combined a pre-procedural formula-based length prediction with limited bedside ultrasound, which was used solely for venous access and to confirm that the guidewire did not enter the IJV—not for tip navigation or tip position confirmation. The catheter insertion length was determined entirely by the formula. Our findings suggest that this combined strategy can serve as a useful first-line safety measure, increasing the likelihood that the catheter is trimmed to a physiologically appropriate length before it enters the patient. However, the formula alone cannot guarantee optimal tip placement, as anatomic variability, respiratory phase, and patient positioning may introduce deviations.

It should be noted that the optimal positioning rate in our study (74.3%) is lower than the rates reported for IC-ECG-guided placement, which generally range from 90% to 97%. This difference is expected, as IC-ECG offers real-time intraprocedural feedback. However, the current formula-based method addresses two fundamentally different clinical scenarios. First, some patients are unsuitable for IC-ECG due to specific factors such as severe arrhythmia, pacemaker dependency, ECMO-related electrical interference, or post-sternotomy anatomy that limits acoustic windows. Second, in many countries and regions, IC-ECG and other advanced navigation systems remain unavailable because of regulatory hurdles, delays in licensing, and limited distribution networks, leaving clinicians without access to real-time tip confirmation regardless of patient factors. In both situations, the formula acts not as a replacement for IC-ECG but as a practical first-line safety measure. Additionally, the mean tip-to-CAJ distance of −0.10 cm and the median of 0.0 cm indicate that even suboptimal placements usually occurred within an acceptable range in the SVC–RA transition zone.

### Comparison with prior bedside length-prediction approaches

Within the AP-CXR family of methods, our findings align with the evolutionary trajectory of predictive accuracy reported in earlier literature. The original regression models, such as those described by Park et al. [14] and later validated in prospective settings by Cho et al. [15], established the utility of using thoracic metrics (e.g., clavicle length, vertebral distance) to estimate PICC length without fluoroscopy. Building on this foundation, Lee et al. presented a refined model that demonstrated a high proportion of optimal tip positioning in controlled test sets [10]. Despite the emergence of real-time navigation as a preferred standard, the continued development of such landmark-based methods by researchers worldwide—including studies by Joshi et al. [5] in India, Zhang et al. [16] in China, and Kim et al. [17] in Korea—underscores a persistent global demand [6,8,16–22]. This trend highlights that advanced systems like IC-ECG are not yet universally accessible due to economic or infrastructural constraints, necessitating reliable anthropometric alternatives for standard wards and emergency settings. By demonstrating operator-independent performance, a median CAJ–tip distance of zero, and very high inter-observer agreement, our results provide a robust external validation of this AP-CXR–based strategy as a precise and dependable alternative when the “gold standard” is technically or economically unfeasible.

### Concomitant intravascular devices

Nearly half of our cohort (45.0%) had additional intravascular devices on CXR (hemodialysis catheters, non-PICC CVCs, ECMO cannulas, chemoports, pacemaker leads). Anatomic and practice guidance consistently favor positioning near the CAJ—rather than in the shallow SVC or the deep RA—to balance flow performance with safety.

For ECMO, Hekmatjah and Kumar reported cases in which PICC or CVC tips became entrapped within the cannula, resulting in massive air embolism [23,24]. In an in vitro study, Franco et al. recommended avoiding placement of a central catheter tip in close proximity to the cannula orifice [12]. Predictable PICC tip localization at the CAJ may reduce—but cannot eliminate—the risk of mechanical interaction with ECMO cannula orifices and other central lines. This is particularly relevant for dual-lumen (DL) internal jugular ECMO cannulas, in which the drainage port may be positioned near the SVC–RA junction, making entrapment of adjacent catheters possible even with appropriate PICC trimming. Clinicians should remain vigilant about potential catheter–device interactions regardless of the pre-procedural length estimation method used. Taken together, the CAJ “safe zone” offers a rational default in wards and ICUs where multiple lines must coexist without fluoroscopic mapping.

For hemodialysis catheters, although direct evidence is limited, if the tip of a high-flow hemodialysis catheter overlaps with that of a PICC, it is reasonable to infer that dialysis efficiency would decline and that fluid replacement or nutritional delivery could also be adversely affected. Ensuring that a PICC tip resides at the CAJ reduces the likelihood of mechanical interaction with dialysis tips that purposely occupy the RA inflow zone.

Although measurement error from AP-CXRs and respiratory phase must be considered—and despite a relatively large SD (2.59)—our real-world validation, showing a mean catheter tip-to-CAJ distance of −0.1 cm and a median of 0 cm, demonstrates the feasibility of tailoring CL to the operator’s intent.

### Limitations

This study had several limitations. First, the single-center, retrospective design using pre-existing radiographs limits generalizability and introduces potential selection bias; prospective, multicenter trials with diverse populations would further validate this approach. Second, AP-CXR inherently carries measurement error due to inconsistent source-to-image distance, making it susceptible to magnification effects. Previous investigators have acknowledged this limitation; however, the approach remains valuable because it uses the primary imaging modality in intensive care and emergency settings where bedside PICC placement is most needed. Third, right-sided approaches predominated (90.9%), reflecting routine clinical practice and limiting statistical power for left-sided analysis. Nevertheless, comparable optimal positioning rates between sides (right 72.5% vs left 69.3%, *p* = 0.590) suggest consistent formula performance regardless of approach side. Fourth, while we acknowledge that IC-ECG is the preferred method for tip location, our study focused on the “unmet need” of bedside placement where such technology is often inapplicable. Future prospective multicenter trials comparing the formula-based approach directly with IC-ECG in ICU settings would further clarify its role. Fifth, the formula relies on fixed skeletal landmarks, such as the carina and vertebral bodies, and does not account for dynamic changes in diaphragmatic position. In patients with significant diaphragmatic displacement—such as those with severe hyperinflation (for example, in acute asthma or a severe COPD exacerbation)—the diaphragm becomes flattened and displaced downward, causing the heart to shift to a more vertical and inferior position. Conditions like abdominal compartment syndrome, massive ascites, or large abdominal masses can also elevate the diaphragm, leading to potential shifts in the CAJ position relative to bony references. Although the original study by Lee et al., which developed this formula, included patients with elevated diaphragms due to ascites and large masses, it did not specifically evaluate populations with extreme diaphragmatic‌‌ displacement. Future research is needed to investigate this further.

## Conclusion

This real-world validation demonstrates that the AP-CXR-based landmark formula achieves accurate and reproducible PICC tip positioning with 97.1% technical success and 74.3% optimal placement. The method showed operator-independent performance across experience levels (1 to >20 years) with excellent interobserver agreement (98.45%) and maintained accuracy regardless of approach side (right 72.5% vs left 69.3%, *p* = 0.590). In complex ICU environments where multiple central lines coexist (45.0% of cases had concomitant ECMO cannulas, hemodialysis catheters, or other devices), consistent CAJ targeting mitigated potential device–device interaction while enabling safe bedside placement without fluoroscopy. These findings suggest that this standardized, landmark-based approach is a useful adjunctive option for bedside PICC placement in critically ill patients who cannot be transferred to angiography suites, although post-procedural radiographic confirmation remains essential.

## Supporting information

S1 File Underlying raw data. This Excel file contains the individual data points used for the statistical analysis of catheter tip locations and operator performance.
(XLSX)
